# Mulberroside A from Cortex Mori Enhanced Gut Integrity in Diabetes

**DOI:** 10.1155/2021/6655555

**Published:** 2021-05-20

**Authors:** Yinyan Xu, Hengli Guo, Tingting Zhao, Jing Fu, Youhua Xu

**Affiliations:** ^1^Department of Pharmacy, Women's Hospital of Nanjing Medical University, Nanjing Maternity and Child Health Care Hospital, Nanjing 210004, Jiangsu, China; ^2^Faculty of Chinese Medicine, State Key Laboratory of Quality Research in Chinese Medicine, Macau University of Science and Technology, Avenida Wai Long, Taipa, Macao SAR 999078, China; ^3^Department of Traditional Chinese Medicine, Zhuhai Maternal and Child Health Hospital, Zhuhai 519000, Guangdong, China

## Abstract

**Background:**

Diabetic endotoxemia has been recognized as one of the hallmarks of type 2 diabetes mellitus (T2DM). Recent findings suggest that gut leak plays a pivotal role in diabetic endotoxemia. Cortex Mori (CM) has been widely applied in China to ameliorate development of T2DM, but its effect on endotoxemia is unknown.

**Methods:**

The study was constructed with two parts: (1) in vivo study of CM on diabetic endotoxemia in db/db mice. Eight C57BL/6 mice were set as normal control; (2) in vitro study of mulberroside A (MBA) from CM on diabetic endotoxemia. Potential mechanism of MBA on ameliorating diabetic endotoxemia was also explored.

**Results:**

The present study found that CM water extract decreased levels of blood glucose, ameliorated liver and renal damage in db/db mice, and ameliorated diabetic endotoxemia (*p* < 0.01). We also found that the water extract enhanced gut integrity and decreased gut inflammatory protein ICAM-1 expression in db/db mice as detected by H&E staining and immunohistochemistry methods. In the in vitro study, MBA decreased levels of MDA and ROS induced by LPS (*p* < 0.01) and enhanced the integrity of gut epithelial barrier (*p* < 0.01).

**Conclusions:**

We found that Cortex Mori and its active component mulberroside A could ameliorate diabetic endotoxemia by preserving gut integrity.

## 1. Introduction

Type 2 diabetes mellitus (T2DM) has now been regarded as an epidemic disease. Although a series of anti-T2DM agents are emerging within this decade, such as GLP-1 analogues [1], this epidemic tendency seems not to be shacked. More importantly, with the prevalent of Western food, this trend is becoming worsened. Because genetic factors should not have dramatic alteration within a few decades, environmental factors must play a role in it. Previously, Hotamisligil and colleagues [2] first demonstrated that neutralizing proinflammatory cytokines helps to ameliorate development of T2DM; since then on, the term “diabetic endotoxemia” is suggested, and “anti-inflammation” has been labeled as one of the indices that evaluate therapeutic efficacy in clinical settings. Within this decade, it is gradually recognized that gut dysbacteriosis might be the source of diabetic endotoxemia [3, 4], as dysbiosis of gut microbiota will contribute to the increase of gut permeability which finally leads to metabolic endotoxemia and higher plasma lipopolysaccharide (LPS) [5, 6]. In this sense, preserving gut integrity is believed to have a beneficial effect on reversing this endotoxemia.

Cortex Mori (CM) is the dried root bark of *Morus alba* L. For centuries, CM has been used in the treatment of diabetes in China. Previously, Qi and colleagues [7] found that CM extract can effectively ameliorate diabetic hyperlipidaemia. A previous study [8] indicated that CM extract concentration dependently suppressed the production of COX-2 and PGE_2_ in vivo and in vitro and showed the anti-inflammatory effects. However, influence of CM on diabetic endotoxemia had not been well investigated.

The current study was constructed with two parts: (1) in vivo study of CM on diabetic endotoxemia in db/db mice and (2) in vitro study of the component from CM (mulberroside A) on diabetic endotoxemia and its potential mechanism.

## 2. Materials and Methods

### 2.1. Materials, Reagents, and Animals

Cortex Mori (CM) was bought from Bozhou Chinese Medicinal Herb Market (Anhui, China). Standard mulberroside A (MBA) was derived from National Institutes for Food and Drug Control of China (Beijing, China). Metformin was purchased from GBCBIO technology (Guangzhou, China). Lipopolysaccharide (LPS) was derived from Sigma (St. Louis, MO, USA). ELISA kits for advanced glycation end products (AGEs) and LPS were purchased from Cheng Lin Biotechnology Company (Beijing, China). Kits for alanine aminotransferase (ALT or GPT), glutamic oxaloacetic transaminase (AST or GOT), HbA1c, and creatinine (Cr) are from Jiancheng (Nanjing, Jiangsu, China). 3-(4,5)-Dimethylthiazo (-z-y1)-3,5-diphenytetrazoli-umromide (MTT), primary antibodies for zona occludens protein-1 (ZO-1), occludin, ICAM-1, p-p38 MAPK, and p38 MAPK were purchased from Santa Cruz (USA). Kit for mAlb was derived from Westang (Shanghai, China). Kits for IL-1*β*, IL-8, MCP-1, and TNF-*α* were purchased from Neobioscience (Shenzhen, China). MDA and ROS detection kits were from Beyotime Biotechnology (Beijing, China). Dulbecco's modified eagle medium (DMEM), fetal bovine serum (FBS), trypsin, MEM nonessential amino acids solution, and L-glutamine were obtained from Gibco (Big Cabin, OK, USA). DAPI, Cy3-, and FITC-conjugated secondary antibodies were supplied by Boster (Wuhan, China). 24-well transwell cell culture plates (hanging insert well diameter 6.5 mm, membrane area 0.3 cm^2^) were obtained from Corning (Corning, NY, USA). The electrical resistance detection system (Millicell ESR-2) was bought from Millipore (Billerica, MA, USA). The other reagents and kits are from commercial sources.

Male C57BL/6 and db/db mice weighing 20–30 g were supplied by Cavens Lab Animal Co. Ltd. (Changzhou, China), and normal chow diet or high-fat diet was, respectively, administrated to the animals. All animal care and investigation conformed to the Guide for the Care and Use of Laboratory Animals published by the US National Institutes of Health (NIH Publication No. 85–23, revised 1996) and was approved by Macau University of Science and Technology. The animals were maintained on a 12/12 h light/dark cycle. Before drug intervention, all animals were kept in animal house for 3 days to make them adapt for the environment.

### 2.2. Preparation of Cortex Mori Water Extract (ECM)

Dry powder of Cortex Mori (CM, 300 g) was immersed with double distilled water (3,000 ml) and continuously boiled on thermostat for 2 h. The water extraction mixture was filtrated and concentrated to 150 ml with the R2002 rotary evaporator, and stock solution for water extract of CM (ECM) was obtained with a concentration of 2 g/ml. The crude drug solution was filtered through a 0.45 *μ*m membrane. Finally, the stock solution was diluted with water to 1.25 g/ml (high dose), 0.58 g/ml (medium dose), and 0.31 g/ml (low dose) and then stored at 4°C until use.

### 2.3. Animals Grouping and Drug Administration

Diabetic animals (db/db mice) were randomly divided into 5 groups as follows: (1) negative control group (*n* = 6); (2) positive control group in which animals were administrated with metformin (0.15 g/kg bodyweight, *n* = 6); (3) low dose Cortex Mori group (0.91 g/kg bodyweight, *n* = 6); (4) medium dose Cortex Mori group (1.82 g/kg bodyweight, *n* = 6); and (5) high dose Cortex Mori group (3.64 g/kg bodyweight, *n* = 6). Eight C57BL/6 mice were set as normal control. Each day, all animals were administrated with drugs as mentioned above or 0.45 ml normal saline (for normal and model group animals) by gavage for 5 continuous weeks. At the end of the experiment, blood samples were collected for biochemical examination, the animals were sacrificed, and colon samples were collected for histological evaluation.

### 2.4. Blood Assay

Blood cell count was measured by the clinical laboratory of Jihua Hospital affiliated to Jinan University (Guangzhou, China). Blood HbA1c, AGEs, AST, ALT, BUN, Cr, LPS, and MCP-1 were determined by kits according to the protocol provided by the manufacturers.

### 2.5. Hematoxylin and Eosin Staining and Immunohistological Evaluation of Colon

Colon was fixed in 4% paraformaldehyde for 24 h and then paraffinized. For observation, sections (0.4 *μ*m) were stained with hematoxylin and eosin (H&E) solution for 5 min, and the histopathological images of colon were observed under a light microscope (Olympus, Tokyo, Japan). For immunohistological evaluation of the colon, sections were first incubated with primary antibody for ICAM-1 (1 : 200); then, biotin-conjugated secondary antibody and streptavidin-biotin enzyme complex were added to the sections to develop brown deposit (positive staining).

### 2.6. Cell Culture

Human epithelial colorectal adenocarcinoma (Caco-2) cell was purchased from the American Type Tissue Collection (Manassas, VA, USA). The cells were maintained in DMEM with 10% fetal bovine serum (FBS), 1% penicillin/streptomycin, 1% MEM nonessential amino acids, and L-glutamine in a cell culture incubator.

### 2.7. MTT Assay

To evaluate influence of LPS and mulberroside A (MBA) on cell viability, cells were treated with MTT (5 mg/ml) for 4 h followed by dimethyl sulphoxide (DMSO) incubation. The absorbance at 490 nm was determined by a spectrophotometer.

### 2.8. Cell Antioxidant Activity Experiment

Cell antioxidant activity was evaluated by determining levels of malondialdehyde (MDA) and reactive oxygen species (ROS) after drugs intervention according to the manufacturer's instruction.

### 2.9. Immunofluorescence Assay

Caco-2 cells were seeded on the slide. After being treated with vehicle, LPS (100 *μ*g/ml), or LPS + MBA (70 *μ*M), cells were fixed with 4% paraformaldehyde. Then, primary antibodies including ZO-1 (1 : 100), occludin (1 : 100), p-p38 MAPK (1 : 100), or p38 MAPK (1 : 100) were added to the cultured cells. DAPI was used for nuclear staining, and FITC- or Cy3-conjugated secondary antibodies were applied to observe proteins' expression or activation under a fluorescence microscope (Olympus, Tokyo, Japan).

### 2.10. Inflammatory-Related Cytokines Assay

Levels of LPS and inflammatory cytokines including IL-1*β*, TNF-*α*, and IL-8 after drugs intervention were determined according to the manufacturer's instruction.

### 2.11. Gut Epithelial Barrier Evaluation

An in vitro gut epithelial barrier model was developed according to our previous report [9]. In general, Caco-2 cells at the density of 1 × 10^5^ were seeded on the upper well membrane of the transwell system and further cultured for 21 days; then, a confluent monolayer was obtained for which transepithelial electrical resistance (TEER) would exceed 400 Ωcm^2^. After drug administration for 24 h, the upper well was added with LPS; finally, level of LPS in the lower chamber of transwell was determined.

### 2.12. Statistical Analysis

The data were expressed as mean ± standard deviation (SD). SPSS 19.0 software with the one-way ANOVA method was applied for data analysis. The *p* value of 0.05 or less was considered to be statistical significant.

## 3. Results

### 3.1. Blood Glycated Proteins

**Figure 1:**
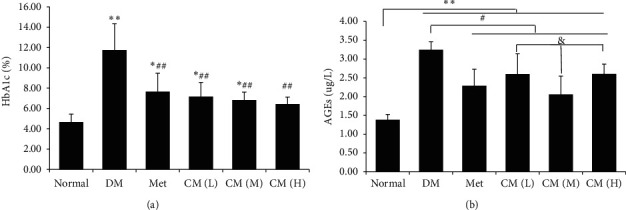
Cortex Mori water extract decreased levels of blood (a) HbA1c and (b) AGEs in db/db mice. DM, diabetic group; Met, metformin group; CM (L), CM (M), and CM (H) indicate diabetic mice administrated with low, medium, and high dose of Cortex Mori water extract. ^*∗*^*p* < 0.05, ^*∗∗*^*p* < 0.01 vs. normal mice; ^#^*p* < 0.05, ^##^*p* < 0.01 vs. DM; ^$$^*p* < 0.01 vs. Met; & *p* < 0.05 vs. CM (M).

High blood glucose is the most significant phenomenon in diabetic population. Long-term exposure of hemoglobin and other forms of proteins to plasma glucose will promote a nonenzymatic glycation pathway and generate glycated proteins, namely, HbA1c or advanced glycation end products (AGEs). Therefore, the level of glycated proteins can reflect average plasma glucose concentration over prolonged periods of time. To identify if oral CM administration could affect this glycation, levels of HbA1c and AGEs were detected by kits. As shown in Figures 1(a) and 1(b), drug administration with either metformin or CM could significantly decrease HbA1c and AGEs levels compared with the diabetic model group, and CM at medium dose had more effect on lowering the AGEs level compared with high or low dose (Figure 1(b)).

### 3.2. Liver and Renal Function

**Figure 2:**
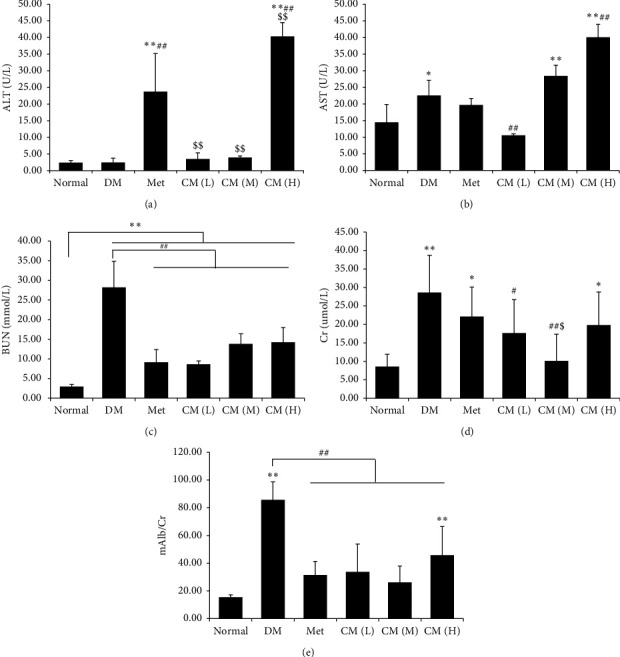
Cortex Mori water extract ameliorate liver and renal damage in db/db mice. Levels of (a) alanine aminotransferase (ALT), (b) aspartate aminotransferase (AST), (c) blood urea nitrogen (BUN), (d) creatinine (Cr), and (e) microalbuminuria (mAlb)/Cr were determined. DM, diabetic group; Met, metformin group; CM (L), CM (M), and CM (H) indicate diabetic mice administrated with low, medium, and high dose of Cortex Mori water extract. ^*∗*^*p* < 0.05, ^*∗∗*^*p* < 0.01 vs. normal mice; ^#^*p* < 0.05, ^##^*p* < 0.01 vs. DM; ^$^*p* < 0.05, ^$$^*p* < 0.01 vs. Met.

To evaluate influence of CM water extract on target organs of diabetes, liver and renal functions were determined. As depicted in Figures 2(a) and 2(b), CM administration at low dose and medium dose would not deteriorate liver function as levels of ALT and AST were not significantly higher than that of the DM group. For renal function, we determined urine BUN (Figure 2(c)), Cr (Figure 2(d)), and ratio of mAlb/Cr (Figure 2(e)). We found that renal function in DM mice was dramatically damaged; in that, all of the three indicators were significantly elevated, while administration with CM at low or medium doses could significantly decrease the renal function indicators.

### 3.3. CM Ameliorated Diabetic Endotoxemia in db/db Mice

**Figure 3:**
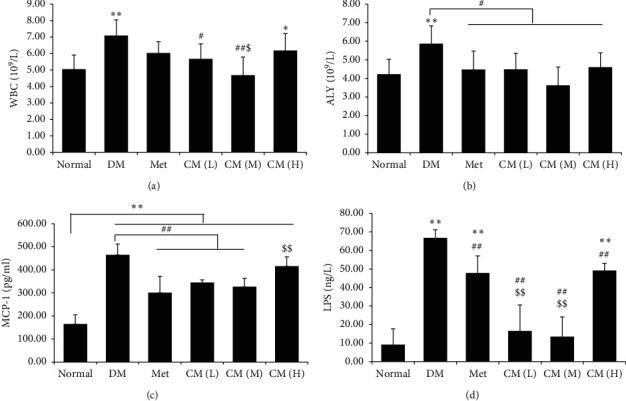
Cortex Mori water extract ameliorated diabetic endotoxemia. Levels of (a) white blood cells (WBC), (b) atypical lymphocytes (ALY), (c) inflammatory cytokine monocyte chemoattractant protein-1 (MCP-1), and (d) lipopolysaccharides (LPS) in blood of db/db mice were determined by kits. DM, diabetic group; Met, metformin group; CM (L), CM (M), and CM (H) indicate diabetic mice administrated with low, medium, and high dose of Cortex Mori water extract. ^*∗*^*p* < 0.05, ^*∗∗*^*p* < 0.01 vs. normal mice; ^#^*p* < 0.05, ^##^*p* < 0.01 vs. DM; ^$^*p* < 0.05, ^$$^*p* < 0.01 vs. Met.

T2DM has now been recognized as a low-grade inflammatory disease, and inflammatory cell infiltration is one of the characteristics [10]. In the present study, we found that the amount of white blood cell (WBC) was dramatically increased compared with normal mice (Figure 3(a)), and administration with either low or medium dose of CM could significantly decrease the level of WBC; more importantly, medium dose of CM possessed more significant effect compared with its low or high dose. We also observed that the number of abnormal lymphocytes (ALY) was significantly increased in diabetic animals, and this elevation could be decreased by any drug intervention in this experiment (Figure 3(b)). The present data suggested that CM at medium dose have a beneficial effect on ameliorating both diabetes and diabetic-inflammatory cell infiltration.

Inflammatory cell infiltration relies on inflammatory chemoattractant protein's expression. MCP-1 is well studied for its role in mediating inflammatory cell infiltration [11]. We observed in the present study that MCP-1 was significantly increased in the diabetic model group compared with normal mice, and administration with metformin or low/medium dose of CM could significantly decrease the level of MCP-1 (Figure 3(c), *p* < 0.01 vs. the diabetic group).

Lipopolysaccharides (LPS), also known as endotoxin, have been found to be elevated in diabetic population and share an important composition in diabetic endotoxemia [12]. In the present study, we found that the serum level of LPS in diabetic mice was increased by about 7 times compared with normal mice (Figure 3(d)); metformin and CM treatment significantly decreased the level of LPS (*p* < 0.01 vs. the diabetic group); more importantly, low or medium dose of CM administration had more significant effect compared with that of metformin on decreasing serum LPS (*p* < 0.01). It is well-known that LPS is an important component of the cell wall in Gram-negative bacteria. In this sense, gut-sourced Gram-negative bacteria (or the so-called bad-microbiota) overproliferation accompanied with gut barrier integrity damage may play a role in diabetic endotoxemia.

### 3.4. CM Preserved Gut Integrity and Decreased Proinflammatory Cytokine's Expression

**Figure 4:**
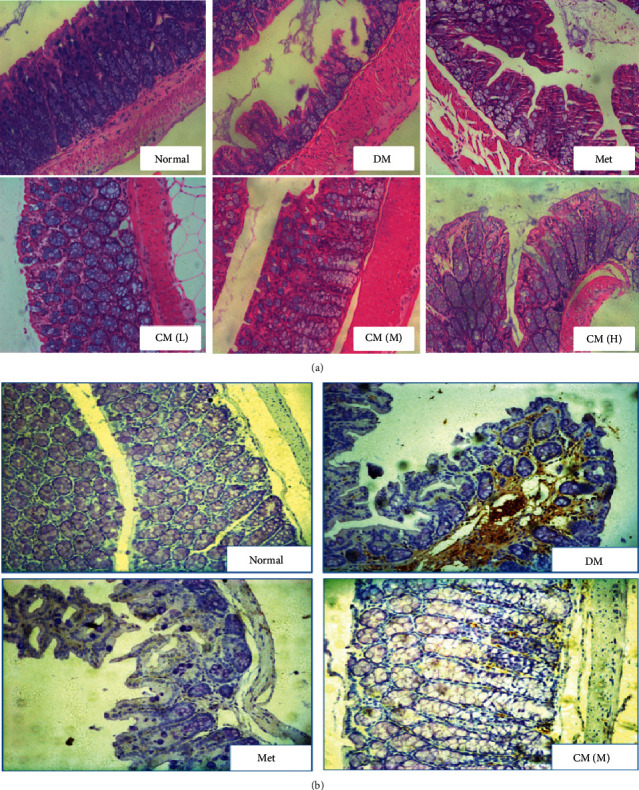
Cortex Mori water extract (a) enhanced gut integrity and (b) decreased gut inflammatory protein ICAM-1 expression in db/db mice as detected by H&E staining and immunohistochemistry methods. DM, diabetic group; Met, metformin group; CM (L), CM (M), and CM (H) indicate diabetic mice administrated with low, medium, and high dose of Cortex Mori water extract. Representative pictures are shown (magnification: 200).

To verify if CM intervention could preserve gut integrity and inhibit “gut-leak” mediated diabetic endotoxemia, we carried out hematoxylin and eosin (H&E) staining and immunohistological evaluation on gut. As shown in Figure 4(a), the gut physical barrier was severely damaged as amounts of gut epithelial cells were detached from the basal lamina; once the animals were treated with metformin or CM, gut integrity was obviously recovered; more importantly, medium dose of CM had more significant effect on preserving gut integrity; in that, this dose administration could restore gut integrity to that of normal state in view of histological evaluation.

As inflammatory cell infiltration was significantly reduced by CM administration at medium dose, we would like to know if any inflammatory protein was involved in this process. ICAM-1 is well recognized to participate in development of diabetes and thereafter inflammatory cell infiltration [13]. To demonstrate our hypothesis, its expression was determined by the immunohistochemistry method. As shown in Figure 4(b), ICAM-1 expression accompanied with inflammatory cell infiltration was dramatically increased in diabetic mice, and medium dose of CM significantly reversed both expression of ICAM-1 and infiltration of WBC. Converging from above findings, we concluded that oral administration with CM had positive effects against diabetic endotoxemia, and the potential mechanism might attribute to its function on preserving gut barrier integrity.

### 3.5. Mulberroside A from CM Enhanced Antioxidative Activity of Caco-2 Cells

**Figure 5:**
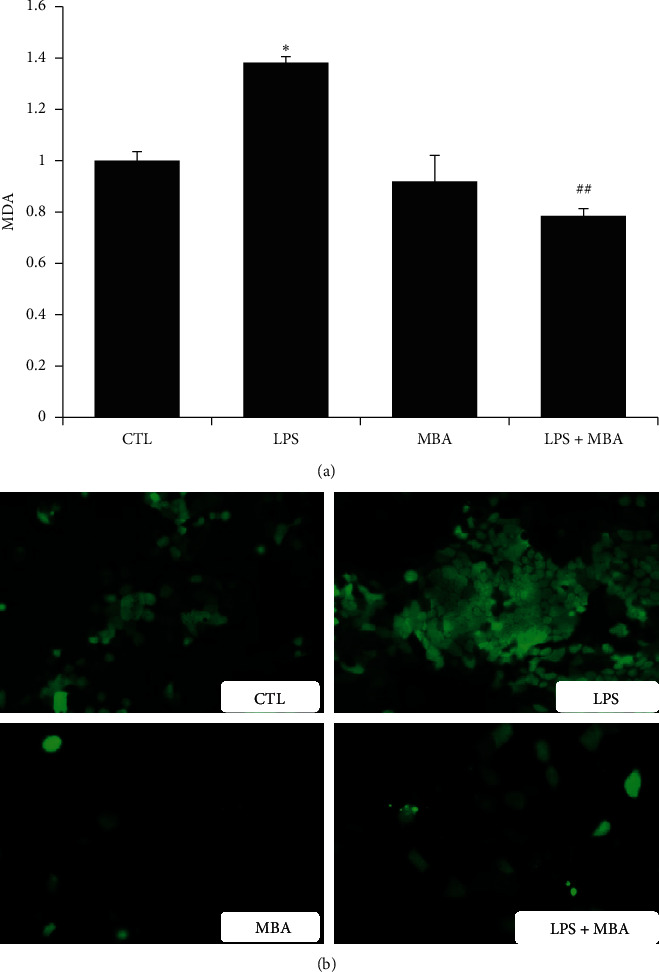
Mulberroside A (MBA) decreased levels of (a) MDA and (b) ROS induced by LPS. CTL, normal control; LPS, lipopolysaccharides group; ROS, reactive oxygen species. ^*∗*^*p* < 0.05 vs. CTL; ^##^*p* < 0.01 vs. LPS.

Mulberroside A is found to be an active component that has been demonstrated to possess multiple activities. Oxidative stress damage is believed to participate in the development of gut leak and diabetic endotoxemia. To investigate if MBA possess some effect on this aspect, Caco-2 cells were incubated with drugs for 24 h; then, the culture supernatant was collected for MDA detection, and the level of ROS in the cells was observed by the immunofluorescence method. As shown in Figures 5(a) and 5(b), LPS stimulation significantly elevated the level of both MDA and ROS in Caco-2 cells, and MBA intervention showed satisfactory effects on decreasing their levels to normal.

### 3.6. MBA Increased Gut Integrity

**Figure 6:**
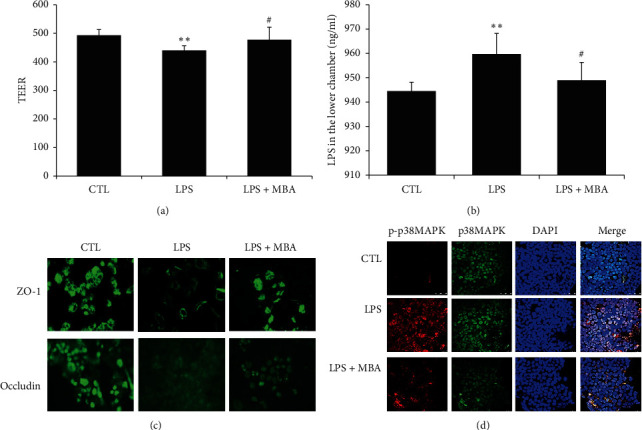
Mulberroside A (MBA) enhanced the integrity of gut epithelial barrier. (a) Transepithelial electrical resistance (TEER) across the monolayers was measured with a Millicell-ERS electric resistance system. (b) LPS was added into the upper chamber, and the permeability of LPS across the barrier to the lower chamber was assayed. (c) Expression of intercellular adhesion molecules proteins including ZO-1 and occludin damaged by LPS in Caco-2 cells was assayed by the immunofluorescence method. (d) Activation of the p38MAPK signaling pathway was studied. Phosphor-p38MAPK was stained by Cy3-conjugated secondary antibody (red), and total-p38MAPK was stained by FITC-conjugated secondary antibody (green); the cell nucleus was stained with DAPI. CTL, normal control; LPS, lipopolysaccharides group. ^*∗∗*^*p* < 0.01 vs. CTL; ^#^*p* < 0.05 vs. LPS.

To evaluate influence of MBA on gut epithelial barrier, we constructed a Caco-2 cell monolayer by a transwell cell culture system. Transepithelial electrical resistance (TEER) across the monolayer was measured with a Millicell-ERS electric resistance system, and the amount of LPS from the upper chamber to the lower chamber across the monolayer was determined. As shown in Figures 6(a) and 6(b), LPS significantly decreased TEER which was accompanied by higher permeability of LPS across the monolayer to the lower chamber (*p* < 0.01), and treatment with MBA strengthened the monolayer integrity.

**Figure 7:**
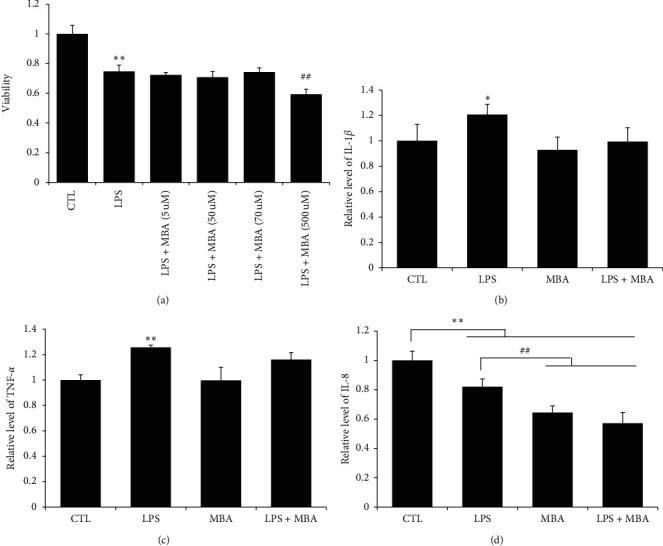
Mulberroside A (MBA) did not influence the viability and inflammation of Caco-2 cells induced by LPS. (a) Viability of cell was determined by the MTT method; levels of (b) IL-1*β*, (c) TNF-*α*, and (d) IL-8 were determined by kits. CTL, normal control; LPS, lipopolysaccharides group. ^*∗*^*p* < 0.05 and ^*∗∗*^*p* < 0.01 vs. CTL; ^##^*p* < 0.01 vs. LPS + MBA (70 *μ*M).

Two pivotal factors including cell viability and tight junction between cells contribute to the integrity of gut barrier [14]. In the current study, we did not find a significant effect of MBA on increasing Caco-2 cell viability (Figure 7(a)), and we would like to know about the tight junction condition after drug administration. In the present study, expression of two tight junction proteins including ZO-1 and occludin was studied. As can be seen in Figure 6(c), these junction proteins are highly expressed on normal Caco-2 cells, and LPS administration strikingly decreased their expression; expectedly, MBA intervention ameliorated this decrease.

To further investigate the potential mechanism, the intracellular signaling pathway activation status was observed. The p38MAPK signaling pathway has been well studied in its role on development of diabetic endotoxemia. As shown in Figure 6(d), the amount of total-p38MAPK in normal, LPS, and LPS + MBA groups were at the similar level. LPS incubation significantly activated p38MAPK, as its phosphor form (p-p38MAPK) was dramatically increased; when the cell was further administrated with MBA after LPS, activation of p38MAPK was significantly inhibited.

## 4. Discussion

Metabolic endotoxemia has now been recognized as a risk factor that is closely accompanied with both the onset and the progress of T2DM [15, 16]. Although there is still a conflict concerning the source of endotoxemia, scholars from both basic research and clinical physician have achieved a consensus that inhibit endotoxemia to help to attenuate the development of T2DM. Many herbal medicines have been used in China to treat diabetes for hundreds of years. However, most of their effecting mechanisms are unknown. In the present study, we investigated influence of Cortex Mori (CM) on diabetic endotoxemia in db/db mice; effects of an active component from it were also evaluated in the in vitro study.

Previously, Hotamisligil and colleagues [2] first demonstrated that neutralizing proinflammatory cytokines helped to ameliorate development of T2DM; since then on, the term “diabetic endotoxemia” is suggested, and “anti-inflammation” has been labeled as one of the indices that evaluate therapeutic efficacy in clinic. Recently, the pivotal role of gut in guarding the body in a healthy status has been more and more recognized. The dysfunction of the gut barrier will result in “gut-leak” and facilitate the entrance of gut-sourced pathogens or toxins into blood circulation; in this sense, if this source is not intervened, it may persistently activate inflammatory cells and enlarge the production of internal-derived inflammatory cytokines, and finally, diabetic endotoxemia occurs. Unfortunately, there is no satisfactory strategy or drug that can solve this problem at present.

Cortex Mori (CM, Sang Bai Pi in Chinese) is the root of *Morus alba* L, which has been traditionally applied to treat diabetic inflammation [17, 18]. To first observe its effect on db/db mice, both hematological and histological experiments were carried out. We found that oral administration of CM water extract at medium dose possessed a significant effect on reducing glycated protein levels (HbA1c and AGEs), ameliorating diabetic nephropathy, and reducing diabetic inflammation in db/db mice. An important finding was that the level of blood LPS was significantly reduced on CM extract application. As LPS cannot be generated from the internal source, we postulated that CM may possess protective effects on inhibiting outsourced LPS entrance into the body. To this end, histological experiment was carried out. By H&E staining, we observed that the gut barrier was severely damaged in diabetic mice, and CM administration significantly preserved its integrity. Moreover, CM administration significantly decreased expression of ICAM-1 and inflammatory cell infiltration in gut wall. Our findings suggest that CM may ameliorate diabetic endotoxemia via enhancing gut integrity. To our knowledge, this is the first report that CM has this effect on gut.

In the present study, we found that low or medium dose of CM possessed protective effects in diabetic mice, while high-dose CM increased serum ALT, AST, creatinine, and LPS levels. We postulated that these adverse effects might be attributed to the over-high-dose CM application. On the one hand, high-dose CM exceeds metabolic ability of the mice and thus induced damage of related organs, e.g., the liver and kidney; on the other hand, oral administration with high-dose CM induced a hypertonic injury in the gut and therefore promoted gut leak and diabetic inflammation. However, exact reason is still waiting to be fully explained.

MBA is one of active components that have been reported to have various activities. To verify if MBA has effects on preserving gut integrity thereafter ameliorating diabetic endotoxemia, a series of in vitro studies were designed. Caco-2 cell is a colon epithelial cell line that has been widely applied to serve as a model to study gut barrier integrity [19]. Previously, we have successfully constructed a monocellular gut barrier model using Caco-2 cells [9]. In the present study, Caco-2 cell line was adopted, and LPS was applied to stimulate Caco-2 cell damage to mimic diabetic gut epithelial barrier damage. Our present in vitro disease model was supported by a previous report from Song and colleagues [20] that LPS administration will induce gut leak accompanied with the epithelial cell loss in mice.

As the epithelial cell loss and inflammation is the characteristic of diabetic endotoxemia, to verify in vitro effects of MBA on LPS-induced Caco-2 cell damaged, the cell viability and inflammatory cytokines secretion were first studied. Report has demonstrated that cytokines including TNF-*α* and IL-1*β* promote the recruitment and infiltration of inflammatory cells and thereafter exaggerate inflammatory damage [21]. Unexpectedly, we did not observe a significant effect of MBA on enhancing cell viability and reversing LPS-induced inflammatory cytokines secretion in Caco-2 cells. So, we conclude from this finding that MBA may not have a direct effect on inhibiting inflammatory cytokines secretion. Oxidative stress is one of the characteristics of diabetes, and excessive oxidative stress may directly induce cell loss which will contribute to gut leak. We noticed that MBA has amounts of “-OH” structure, and we postulate it may possess some effect against oxidative stress. Fortunately, we found that MBA has definite effects against LPS-induced oxidative stress by reducing the level of MDA and ROS within cells.

As discussed above, gut leak will result in the paracellular invading of luminal antigens and toxins into blood circulation [22, 23]. Recent understanding depicts the importance of gastrointestinal tract leaky barrier in the development of DM [24]. The tight junction between intestinal epithelial cells is a natural barrier against invasion of intestinal toxins and bacteria into blood circulation [25, 26], and cell quantity and tight junction proteins among cells are two important factors that ensure gut barrier integrity [19, 27]. As we did not observe, MBA had a protective effect on gut epithelial cell viability, and we would like to know if it has some effects on expressions of tight junction proteins. We found that MBA administration significantly elevated expression of tight junction proteins expression, including ZO-1 and occludin. Our present finding is in accordance that LPS administration can decrease tight junction proteins' expression and increase the paracellular permeability [20]. Our finding was further verified; in that, the permeability of LPS across gut epithelial barrier was decreased, and TEER was enhanced on MBA administration.

As discussed above, endotoxemia has been found to be one of pivotal factors that promote development of diabetes, and gut leak might be a new target on preventing disease progression [15, 16]. In the present study, we found oral administration with the extract of Cortex Mori decreased the serum level of LPS and preserved integrity of gut barrier. Therefore, we detected the underlying mechanism in Caco-2 cells. Previously, Koistinen and colleague found the p38 MAPK signaling pathway contributed to insulin resistance in skeletal muscle cells [28]. Besides that, p38 MAPK has also been demonstrated to be the pivotal cell signaling pathway that mediates both inflammation and tight junction proteins expression. It is reported that activation of p38 MAPK will increase inflammatory cytokines expression and secretion, while it will decrease expression of tight junction proteins among epithelial cells [29, 30]. To further investigate its role in MBA-mediated function in gut barrier integrity, its activation was studied by the immunofluorescence method. We found that LPS significantly decreased TEER and activated p38 MAPK, while MBA administration significantly reduced the level of p-p38 MAPK and increased gut barrier integrity in Caco-2 cells.

In conclusion, we demonstrated in the present that oral administration with Cortex Mori water extract could ameliorate diabetic endotoxemia by enhancing gut integrity. By in vitro study, we demonstrated that MBA had a significant effect against LPS-induced gut epithelial barrier damage, anti-inflammation, antioxidative stress, and enhancing tight junction proteins expression involved in its protective effect, and p38MAPK participated in this process. Our present findings may supply novel idea on R&D of antidiabetic drugs from Chinese medicines.

## Data Availability

The datasets used to support the findings of this study are available from the corresponding author upon request.
